# Study of 3D-printed chitosan scaffold features after different post-printing gelation processes

**DOI:** 10.1038/s41598-018-36613-8

**Published:** 2019-01-23

**Authors:** Carlo Bergonzi, Antonina Di Natale, Francesca Zimetti, Cinzia Marchi, Annalisa Bianchera, Franco Bernini, Marco Silvestri, Ruggero Bettini, Lisa Elviri

**Affiliations:** 1 Food and Drug Department, University of Parma, Parco Area delle Scienze 27/A, 43124 Parma, Italy; 2 Interdepartmental Centre Biopharmanet-Tec, University of Parma, Parco Area delle Scienze 27/A, 43124 Parma, Italy; 3 Department of Engineering and Architecture, University of Parma, Parco Area delle Scienze 181/A, 43124 Parma, Italy; 4 Department of Innovative Technologies, University of Applied Sciences and Arts of Southern Switzerland (SUPSI), CH-6928 Manno, Switzerland

## Abstract

3D biomaterial manufacturing strategies show an extraordinary driving force for the development of innovative therapies in the tissue engineering field. Here, the behaviour of 3D printed chitosan (CH)-based scaffolds was explored as a function of the post-printing gelation process. To this purpose, gel forming properties of different media were tested on their capability to retain 3D structure, water content, mechanical resistance and surface/internal porosity. Three different gelation media (i.e. KOH 1.5 M, Na_2_CO_3_ 1.5 M, ammonia vapours) were selected and the 3D CH scaffolds were tested in terms of biocompatibility toward fibroblast as skin associated human cell line.

## Introduction

A serious impairment in skin tissue or in other organs can be mainly due to traumatic events or by pathological alterations that lead to low availability of nutrients. The extra-cellular matrix (ECM) plays a primary role in the regeneration process, since its function consists in coordinating cell proliferation, spatial orientation, differentiation and maturation. ECM provides characteristics of storage and delivery of growth factors and cytokines and it supplies structural integrity and scaffolding features^1^ as well as a *substratum* for molecules such as glycosaminoglycans, hyaluronic acid and collagens, naturally secreted by recruited cells during initial phases of regeneration.

One of the purposes of tissue engineering is that of developing synthetic or naturally-derived biological substitutes (scaffolds) capable to help injured tissues to heal properly.

Chitosan has been widely studied for decades targeting several potential application fields as, for instance, the biopharmaceutical one, that has been of increasing interest throughout years for its remarkable and promising biological and biomechanical properties, mainly explained by its unique polycationic character^2,3^. Furthermore, chitosan exploitability for medical purposes is sustained by the proofs of its biocompatibility and nontoxicity^4^ as well as FDA approval^5^. Most of applications and research studies on chitosan focus on its hydrogel state. Chitosan is soluble in acid aqueous media at pH 5–6 at 24 °C; sol-gel transition can occur by known gelation techniques such as ionotropic, cross linker-mediated, polyelectrolyte complexed or self-assembled^6^. Each one provides different characteristics to the gel material formed; cross linking is often performed with glutaraldehyde, commonly used in protein complexation, that causes the formation of rigid irreversible textile, resistant to very-low pH’s and to traction mechanical stress. Anyway, aldehydes frequently result toxic for mammals and in particular for human tissues and it’s also reported they can have a pollutant environmental impact^7^, for these reasons their use for *in vitro* or *in vivo* application is usually avoided.

Polyelectrolyte complexes formation can be exploited for the introduction in the system of other polymers such as gelatin, alginate or hydroxyapatite. Ionotropic gelation is based on the principle that negatively charged ions electrostatically interact with the cationic groups of the polymer chains, enabling the formation of hydrogen bonds, necessary to form a “rigid” but flexible network. The most commonly used substances include citrates, phosphates, sulphates, TPP or other alkaline media. The size and nature of negative ions, their concentration, their contact time with the polymer and pH can systematically influence its swelling behaviour in conjunction with its rehydration capacity. Mechanical properties as well in terms of elastic modulus (Young’s modulus) and toughness can be influenced by the type of gelation; in general, the higher is the number of interactions formed, the stiffer is the hydrogel obtained.

Physical cross-linked chitosan hydrogels can be prepared, avoiding the employment of toxic solvents or catalysts, by putting into contact chitosan solution with a basic agent, in liquid or gaseous state. Since bases that are used are water soluble and can be washed away by simply rinsing with water, resulting gels have a far least hazardous profile when in contact with the human body and as a consequence they are more suitable and easy to manage for pharmaceuticals. The reaction of gelation without the use of organic solvents is traditionally performed with the help of strong bases such as KOH or NaOH^8^ this from one side guarantees a fast and complete gelation but, on the other side this limits the control and the fine tuning of swelling behaviour, porosity and of main mechanical properties such as strength and elasticity^3^.

Another issue associated with the use of strong bases as gelation agents is the potential chemical degradation of any active compound included in the formulation: actually it is very desirable to use hydrogels also as storage or delivery systems of molecules^9^, such as growth factors or drugs, and this urges the need for milder conditions of gelation, in order to avoid the chemical degradation of those substances during the manufacture process.

In an interesting work by Xu and coworkers^10^, the gelation of chitosan gels under mild conditions was reported by using NaCl or phosphate buffer saline as gelling solutions. Authors described a process that required a very long time of gelation and resulted in gels that dissolved completely within 24 or 48 hours. In our opinion this time is not adequate in view of a possible application of the product as medical device, so a first aim of this work was to propose and compare different mild gelation conditions in order to obtain hydrogels in a shorter time, namely not over 2 hours, having a longer persistence in biological buffers as well as being suitable for cell culture. Scaffold duration also depends on their mechanical properties, which can be modulated by the type of gelation agent used. Knaul *et al*.^11^ described the production of chitosan fibres by wet spinning and reported the improvement of their mechanical properties by immersing them in a NaOH solution buffered with KH_2_PO_4_ at pH 5.4: they explained this behaviour by the formation of a cross-linked network generated by the bridging effect of phosphate ions between chitosan molecules.

On the basis of these data, in this work chitosan scaffolds were prepared from formulations already developed and studied for tissue engineering purposes^8^ in different cell culture systems^12,13^. With the perspective of a serial production, the manufacture of chitosan scaffolds was automated with the help of a dedicated 3D printer for freeze-deposition. The polymer is extruded on a cooled surface, causing its instantaneous freezing before undergoing the controlled ionotropic gelation process. This offers the chance to design *in silico* the microarchitecture of scaffolds as well as to guarantee high repeatability. This results in a significant improvement in control of pore size both at macroscopic and microscopic level, by creating *ad hoc* geometries to favour cell adhesion and growth with respect to traditional casting techniques^14^. Cryogenic 3D printing for hydrogel modelling based on different approaches (i.e. use of liquid nitrogen, cooled surfaces, dry ice) was successfully used for different inks (polyvinyl alcohol, Phytagel, etc.) demonstrating its ability to create scaffold mimicking soft tissues^15–17^.

When chitosan is used, the structure deposed by 3D printing and frozen is then subjected to gelation which can significantly affect the final characteristic of the scaffold: for this reason, in this paper, different alkaline agents, salt concentration and times of gelation were investigated in order to identify which seem to be more promising for an accurate control of the aforementioned chemo-physical features, which respond better to *in vitro* biocompatibility tests.

## Results

In this paper we addressed the role of the gelation medium on the physical, mechanical and biological characteristics of chitosan scaffolds prepared by 3D deposition and freezing.

The sol-gel transition of chitosan solutions is determined by the contact with basic media^18^. Having this in mind, and with the target of screening gelation conditions suitable to retain reproducible and accurate 3D printed structure, we took into consideration a gas, ammonia vapours, and a series of aqueous solutions prepared from a strong base such as KOH, at decreasing concentrations, or weak bases such as carbonate or phosphate salts alone or in association. The neutralization process was visualized by the observation of colour changes in scaffolds printed from a chitosan solution to which blue bromothymol was added as a pH indicator (see Methods) solutions (Figure S1, Supplementary Material). Solutions that required more than one minute to fix the structure were excluded, since this time was considered not compatible with the time of melting of 3D frozen scaffolds. In general, solutions with a pH inferior to 11 were not able to fix and maintain the structure of scaffolds (Table 1).

**Table 1:** The effects of chitosan gelation media, solution concentration, pH on the maintenance of 3D printed structure (gelation process carried out at room temperature).

Gelation media	Concentration	pH	Maintenance of 3D structure	Neutralization time (sec)
KOH	1,5 M	14	✓	30
	1 M	14	✓	50
	0,5 M	13.70	✓	60
	0,1 M	13.13	✗	300
Na_2_CO_3_	1,5 M	11.94	✓	40
	1 M	11.74	✓	50
Na_2_CO_3_+NaHCO_3_	0.5 M 0.5 M	9.72	✗	90
Na_2_CO_3_+NaHCO_3_	0.01 M 0.09 M	9.22	✗	n.d.^*^
Na_2_CO_3_+NaHCO_3_	0.05 M 0.05 M	10.05	✗	240
Na_2_HPO_4_ K_2_HPO_4_	0.1 M 0.005 M	8.08	✗	n.d.^*^
Na_2_HPO_4_+Citric acid	0.1 M 0.05 M	7.6	✗	n.d.^*^
Ammonia vapour from 28% aqueous solution		13	✓	18

^*^n.d. = not detectable.

The addition of NaCl, ranging from 0.05 to 0.5 M, did not accelerate the time of gelation or rather resulted in a consistent increase in time of gelation with loss of 3D structure (data not shown): this is reasonably due to the acceleration of melting of the structure due to freezing-point depression.

**Figure 1:**
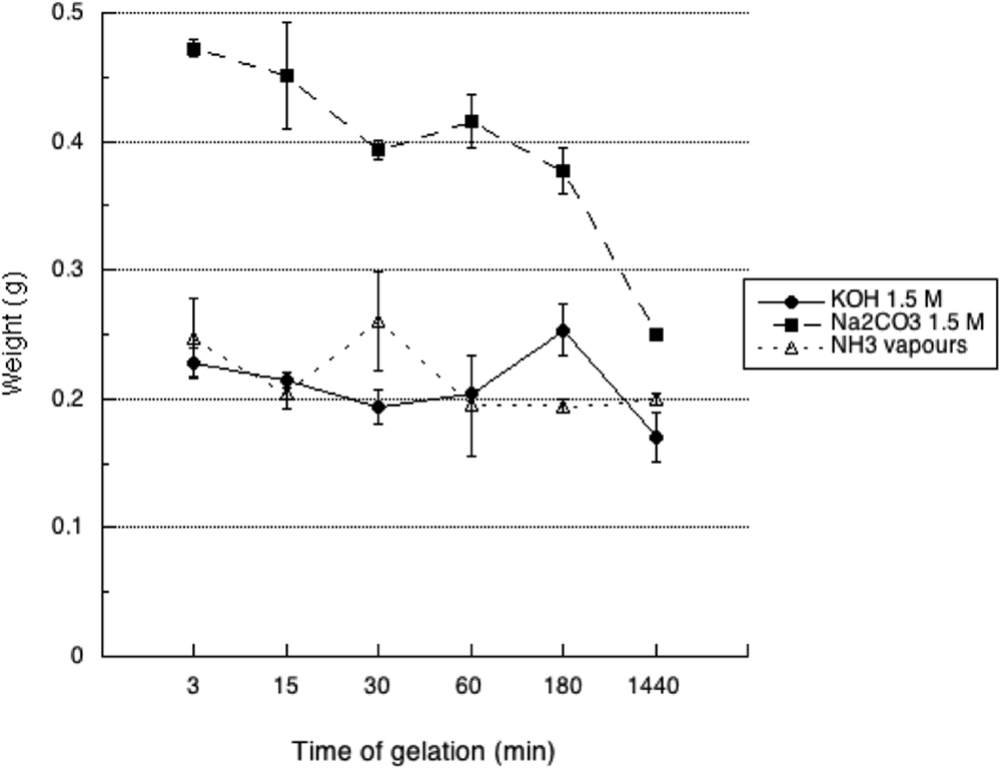
Measured wet weight (W_0_) of 3D printed CH-based scaffold gelled with KOH (1.5M), Na_2_CO_3_ (1.5M), NH_3__(g)_ (from 28% ammonia solution) as a function of time.

Further experiments were performed on gelation media that guaranteed maintenance of 3D structure and neutralization: in particular a further selection was executed on the basis of neutralization time. These tests lead to the selection of three gelation media, namely KOH 1.5 M, Na_2_CO_3_ 1.5 M and ammonia gas, which showed a complete neutralization of solutions by 2 minutes. To confirm this observation, and to verify the real completeness of gelation, test on weight and water content were performed after different time of exposition to gelation media. Surface water was removed as described in Methods section, then W_0_ was recorded (Fig. 1). At first time point, the weight of scaffolds immersed in Na_2_CO_3_ was almost twice the weight of the other two types of scaffolds (Na_2_CO_3_ 0.3412 g ± 0.0122 vs KOH 0.1742 g ± 0.0054 g; ammonia vapours 0.1688 g ± 0.0083 g). While the weight of scaffolds prepared with KOH or ammonia gas did not vary significantly at all time points (mean weight in KOH 0.1768 g ± 0.0131 g; in ammonia vapours 0.1720 g ± 0.0185 g; paired t test, p < 0.05), the weight of scaffolds prepared with Na_2_CO_3_ decreased with time and did not stabilize until 24 hours (Fig. 1). Even if for Na_2_CO_3_ group a longer time could give a more consistent gelation, the time of 1 h was chosen as optimal time for all conditions tested, also considering a possible translation of the method in a production environment.

Resolution of the structure is dependent on the 3D printing procedure and was reported on a previous work published by Elviri *et al*.^14^. Starting from the CAD design, the theoretical pore size was 200 μm while nominal filament diameter was 250 μm. After 1 h gelation, at the maximum swollen state, the filament size was measured (n = 9) depending on gelation media. Actually, for KOH mean filament size was 273 ± 28 μm, for Na_2_CO_3_ mean filament size was 293 ± 28 μm, whereas for NH_3_ vapours mean filament size was 259 ± 29 μm.

**Figure 2:**
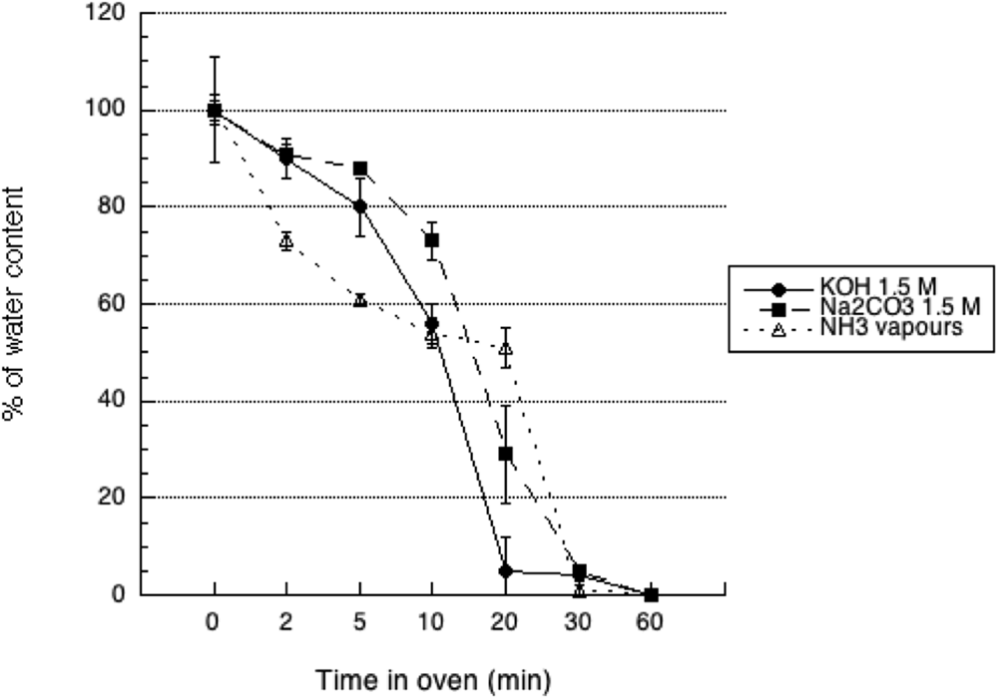
De-hydration profile of 3D CH-based scaffolds gelled with KOH (1.5M), Na_2_CO_3_ (1.5M), NH_3(g)_ (from 28% ammonia solution) as a function of time. Scaffolds were exposed to vacuum oven at 40 °C until complete loss of water.

After one hour of gelation and 20 seconds of water removal by vacuum on filter paper the weight of scaffolds was significantly different depending on the method of gelation. In particular, gelation in Na_2_CO_3_ gave the highest weight, followed by KOH then ammonia vapours. After lyophilisation all types of scaffolds reached a constant weight (p < 0.05) after one hour of drying but the rate of water loss was different on the basis of the gelation media (Fig. 2). Scaffolds prepared in KOH showed the steeper rate of drying, followed by those prepared in Na_2_CO_3_. Scaffolds prepared in ammonia vapours showed an interesting two-phases loss of water reaching a plateau after 10′ of drying, when the amount of left water was about 55%, followed by a second dehydration phase that lead to complete drying.

The complete dehydration of scaffolds resulted in their deformation: the original morphology was lost and was not recovered even after immersion in water for more than 24 hours. Very similar volumetric decrease was observed (n = 9) for the three gelation media: 45.7% for KOH, 43.0% for Na_2_CO_3_ and 41.7% for NH_3_ (RSD < 2%).

**Figure 3:**
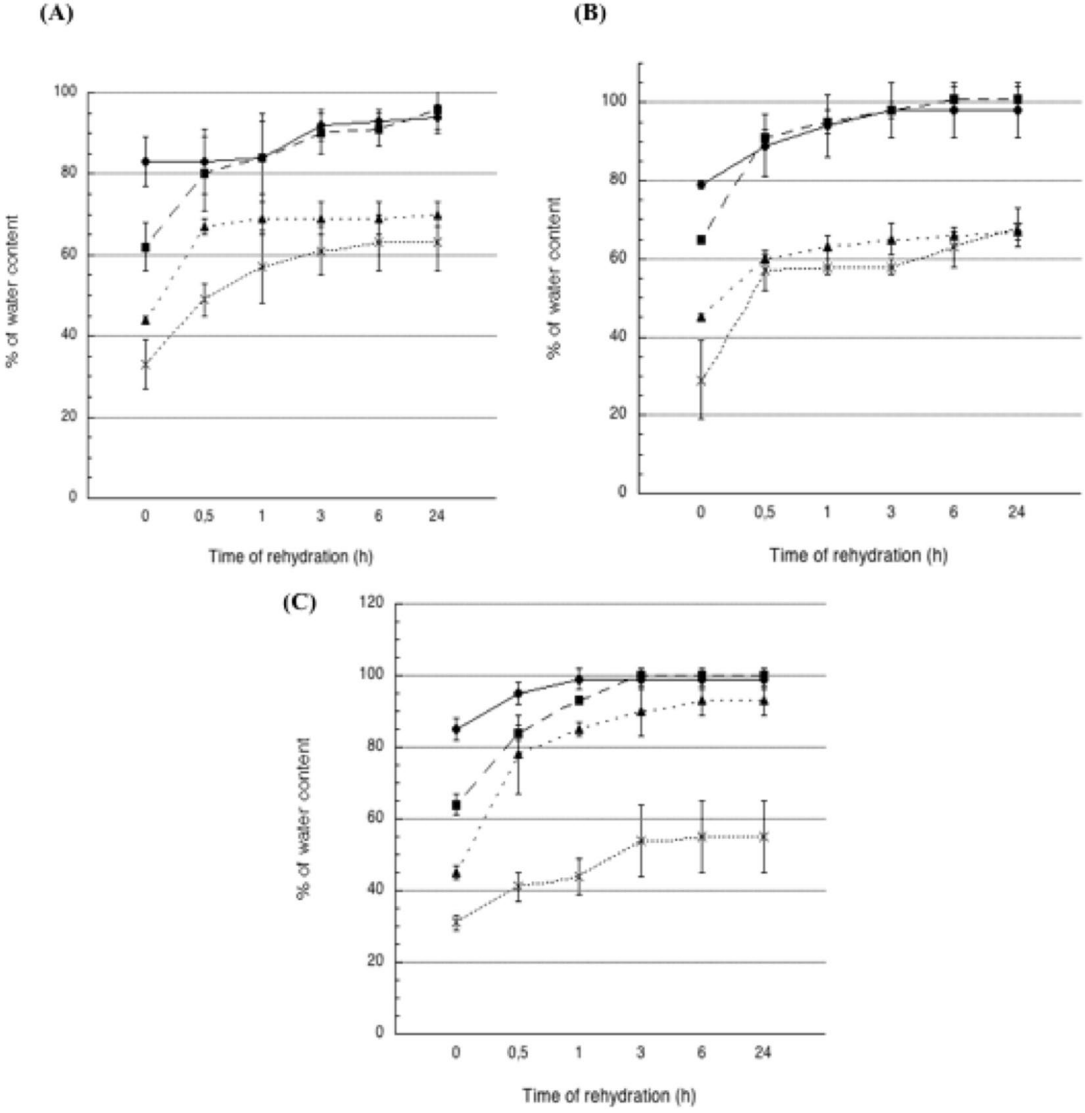
Re-hydration profile of 3D CH-based scaffolds previously de-hydrated at different percentages and left in water as a function of time. Scaffolds were gelled in (**A**) KOH (1.5M), (**B**) Na_2_CO_3_ (1.5M), (**C**) NH_3(g)_ (from 28% ammonia solution) and exposed to vacuum oven at 40 °C at different times to obtain the desired lost percentage of water, namely 30% (cross), 45% (solid triangle), 60% (solid square) or 85% (solid circle) of initial water weight.

In order to test to which extent the drying could be pushed to get the complete recovery of original shape and water content, on the basis of the drying experiment described above, we selectively dried scaffolds to 80%, 60%, 45% and 30% of their starting weight. Those scaffolds were immersed in ultrapure water and their weight was checked at regular intervals up to 24 hours (Fig. 3). Scaffolds exposed to KOH failed in recovering their initial weight, whatever the starting dehydration state. This was probably due to the fact that in KOH gelation occurred suddenly, leading to a rapid fixation of chitosan hydrogel in the shape given by 3D printing, preventing the set up of new interactions between chitosan chains and water. The ability to recover water was more and more hampered as the drying was pushed further: actually, while scaffolds dried at 80 or 60% of starting weight returned to the 95% of their starting weight after 24 hours, scaffolds dried at 45 or 30% of starting weight did not go beyond the 70% of weight recovery in 24 hours. On the other hand, scaffolds prepared in Na_2_CO_3_ recovered their starting weight in about 3 hours, provided that hydration state was not below 60%. On the contrary, scaffolds dehydrated to 45 or 30% did not go beyond the 68% of weight recovery after 24 hours of immersion. Also scaffolds prepared in ammonia vapours regained their starting weight in 3 hours if the starting hydration state was at 80 or 60%. Water recovery was at 93% for scaffolds starting from a 45% water content, while scaffolds dehydrated to 30% only reached a 55% of weight recovery.

DSC analysis showed that scaffold gelled in Na_2_CO_3_ showed the highest free water value of 96.78 ± 2.47%, followed by those gelled by exposure to NH_3_ vapours reaching a value of 94.44 ± 2.42% whereas gelation by immersion in the KOH solution exhibited the lowest value of 88.57 ± 4.23%. Interestingly, it seems there is a correlation between the pH of the gelling conditions (Na_2_CO_3_ pH 12; NH_3_ vapours pH 13; KOH pH 14) and the amounts of bulk water in the samples, suggesting that the stiffer is the network at the molecular level, due to a stronger ionotropic gelation, the more water is bound to the polymer.

Young’s modulus was calculated from the linear portion of the stress-strain curve (Figure S2 Supplementary Material) and resulted respectively for KOH 105 kPa ± 18 kPa (% strain at rupture 34,6% ± 2,4%), for Na_2_CO_3_ 94 kPa ± 19 kPa (% strain at rupture 43,9% ± 9,2%) and for ammonia vapours 128 kPa ± 21 kPa (% strain at rupture 52,3% ± 5,3%). For all the scaffolds, the stress-strain curve presented only a linear shape, with the elongation directly proportional to the applied stress. The scaffolds behaved elastically when subjected to stresses, and the application of an additional stress did not lead to further alterations of the hydrogel’s mechanical properties (Figure S2 Supplementary Material).

No statistical difference existed between hydrogels prepared in KOH or Na_2_CO_3_, while scaffolds prepared in ammonia vapours showed a significantly higher elastic modulus with respect to both other media.

**Figure 4:**
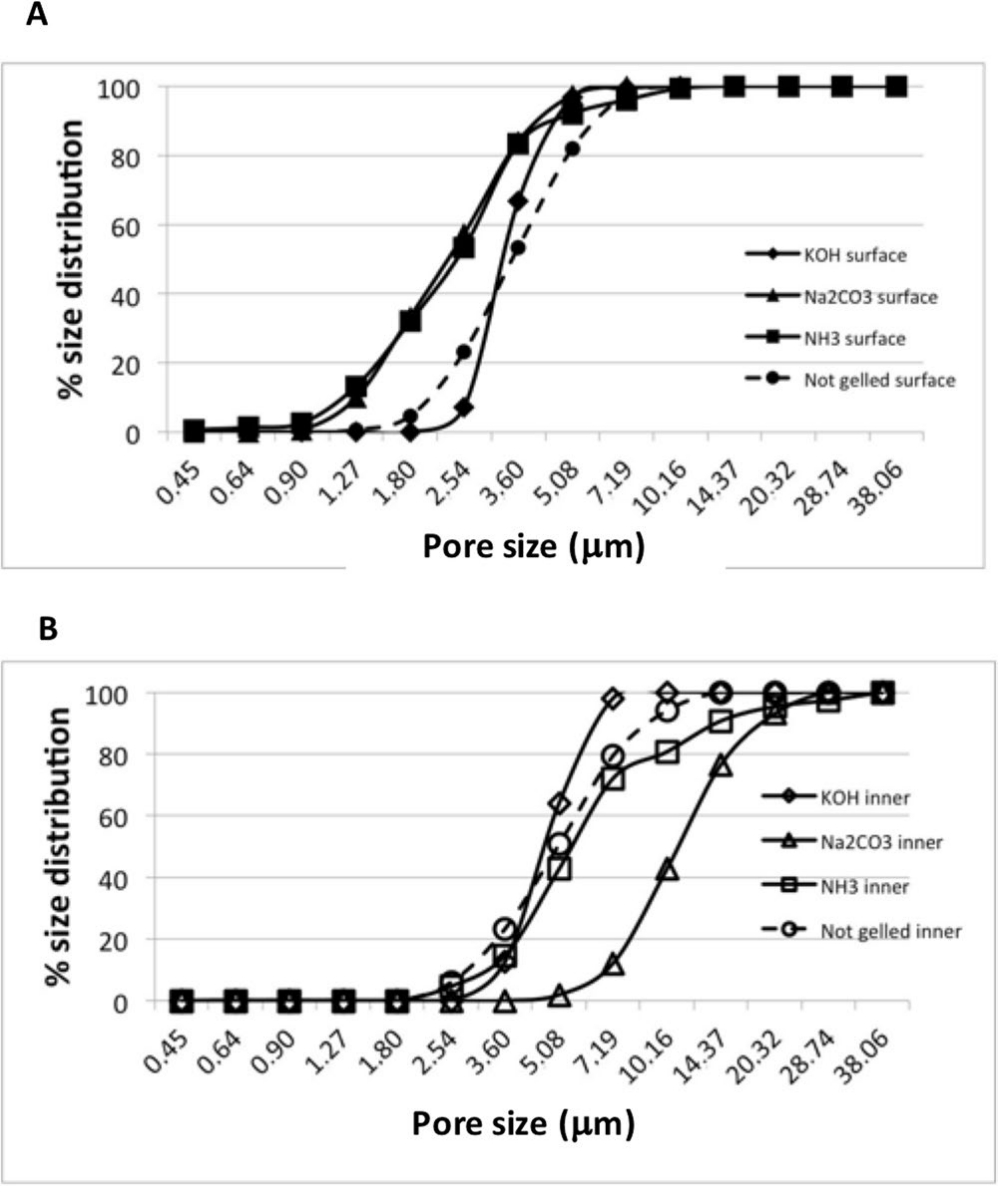
(**A**) Surface and (**B**) inner pore size distribution percentage measured by SEM of 3D printed CH-based scaffolds gelled with KOH (1.5M), Na_2_CO_3_ (1.5M), NH_3(g)_ (from 28% ammonia solution).

In an effort to explain the reason of such a different stiffness, we analysed internal morphology of scaffolds by SEM. Pictures of surfaces and transversal sections of hydrogel filaments were taken and pore size and distribution were measured, the appearance of surface is quite different on the basis of gelation media (Figure S3, Supplementary Material). Hydrogels prepared in KOH show a rough surface with pores having a mean size distribution ranging from 2 to 9 μm. Scaffolds prepared in Na_2_CO_3_ have a mean pore size diameter distribution of 1–7 μm. Hydrogels prepared in ammonia vapours show pores that span in a wider range, from 0.5 to 13 μm. No correspondence is observed with internal porosity, that is between 3 and 14 μm for scaffolds prepared in KOH, 5 and 39 μm for scaffolds prepared in Na_2_CO_3_ and between 2 and 42 for those prepared in ammonia vapours (Fig. 4). Differences in surface appearance reflect the differences in the mechanism of gelation and could explain as well the differences in the ability to reuptake water after dehydration.

The ATR FT-IR spectrum of raw chitosan was compared with spectra of hydrogels prepared with the selected gelation media (Figure S4, Supplementary Materials). The transmittance spectrum of hydrogels prepared with KOH showed a broad widening of the O-H stretching band^19^, masking the stretching vibration of N-H. In hydrogels prepared with Na_2_CO_3_ or ammonia vapours, the band was slightly shifted to higher wavenumbers, and the stretching vibrations of O-H or N-H were undistinguishable as well.

In KOH hydrogels only, the stretching vibration of the C-H attributed to the pyranose ring was smoothened with respect to raw material (2877 cm^−1^), and slightly moved to higher wavenumbers (2886 cm^−1^), while no differences were observed between raw materials and hydrogels prepared with Na_2_CO_3_ or ammonia vapours (2879 cm^−1^ and 2874 cm^−1^). On the other hand, the bending of the C=O bond was slightly shifted to higher wavenumbers in hydrogels prepared in Na_2_CO_3_ or ammonia vapours (1655 cm^−1^ and 1653 cm^−1^) with respect to raw material (1638 cm^−1^), while the peak was maintained at 1636 cm^−1^ in hydrogels prepared with KOH.

In hydrogels prepared with KOH, the peak at 1570 cm^−1^ observed in raw material (probably attributable to N-H bending vibration in amide group^19^) was smoothened and almost disappeared, while it was preserved with almost the same intensity in the two other types of hydrogels prepared with Na_2_CO_3_ and ammonia vapours. In the fingerprint region, the symmetrical bending of C-H at about 1374 cm^−1^ was not affected by gelation. The region between 1160 cm^−1^ and 1025 cm^−1^, derived from different stretching vibrations of C-O bond is the more affected one: with respect to raw material, the transmittance in the KOH sample is increased, with a shift of the peak at 1074 cm^−1^ to 1080 cm^−1^ and a shift of the signal at 1026 cm^−1^ to 1035 cm^−1^ due to the stretching of C-O. In the Na_2_CO_3_ and ammonia vapour hydrogels, the transmittance of the 1074 cm^−1^ band increased with respect to raw material.

These data support the involvement of evidenced groups in the formation of hydrogen bonds that sustain the structure of hydrogels. The differences observed among the scaffolds could be attributed to the different distribution of water molecules occurring during the polysaccharide cross-linking process. In particular, scaffolds prepared with ammonia or Na_2_CO_3_ have very similar content of free water and presented almost overlapped ATIR spectra with respect to scaffolds prepared in KOH.

**Figure 5:**
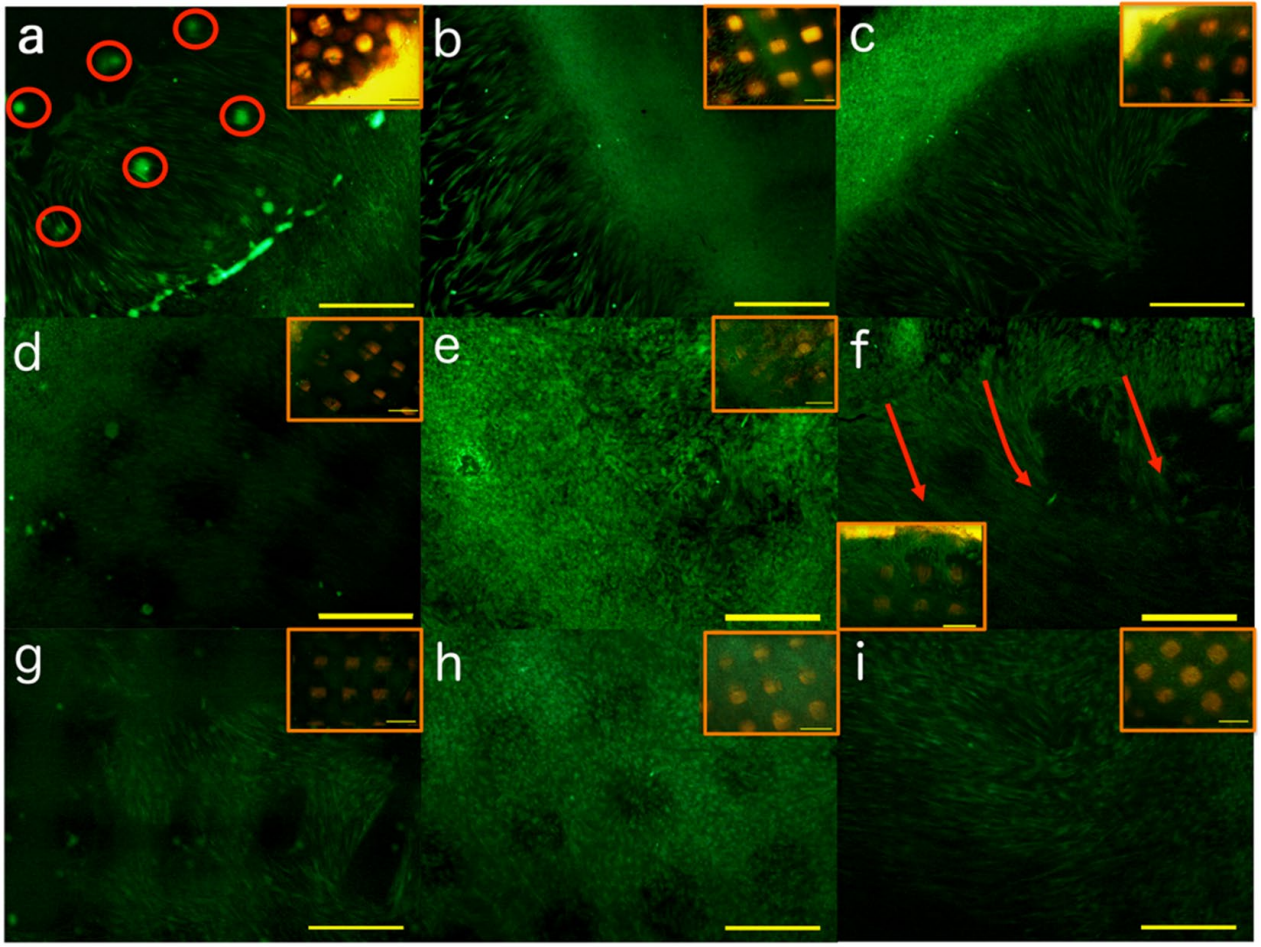
Fluorescence microscopy images of calcein AM-stained fibroblasts grown on hydrogels prepared in KOH 1.5M (**a**,**d**,**g**), Na_2_CO_3_ 1.5M (**b**,**e**,**h**) or NH_3(g)_ (from 28% ammonia solution) (**c**,**f**,**i**). All images are at magnification 4 X O.M.; scale bars 500 μm). (**a**,**b**,**c**) cells grown for 4 days migrating from the plate onto chitosan scaffolds. Red circles indicate cells grown in clusters into macropores; (**d**,**e**,**f**) cells grown for 14 days. Red arrows indicate the direction of cell migration on the scaffold. (**g**,**h**,**i**) cells grown for 21 days. In this day differences in cell growth on different scaffolds are less appreciable from microscopic observation. Images in inserts show the picture of the same field taken by contrast phase microscopy, showing the position of scaffolds with the grid structure conferred by 3D-printing.

Human fibroblasts were cultivated on chitosan scaffolds prepared with KOH, Na_2_CO_3_ or ammonia vapours and their growth efficiency was compared. Living cells were checked during the whole experiment by periodically adding calcein AM to cultures and by observing them under fluorescent microscope. Images after 4 days confirmed that all scaffolds supported cell growth: cells that at the moment of seeding fell inside macropores created by scaffold design (see arrows in Fig. 5a) grew in clusters, while cells that initially adhered to plate migrated on the scaffold, covering it almost completely in 14 days. In Fig. 5f it is possible to appreciate that the direction of cell migration was driven by the orientation of filaments composing the grid of the hydrogel.

**Figure 6:**
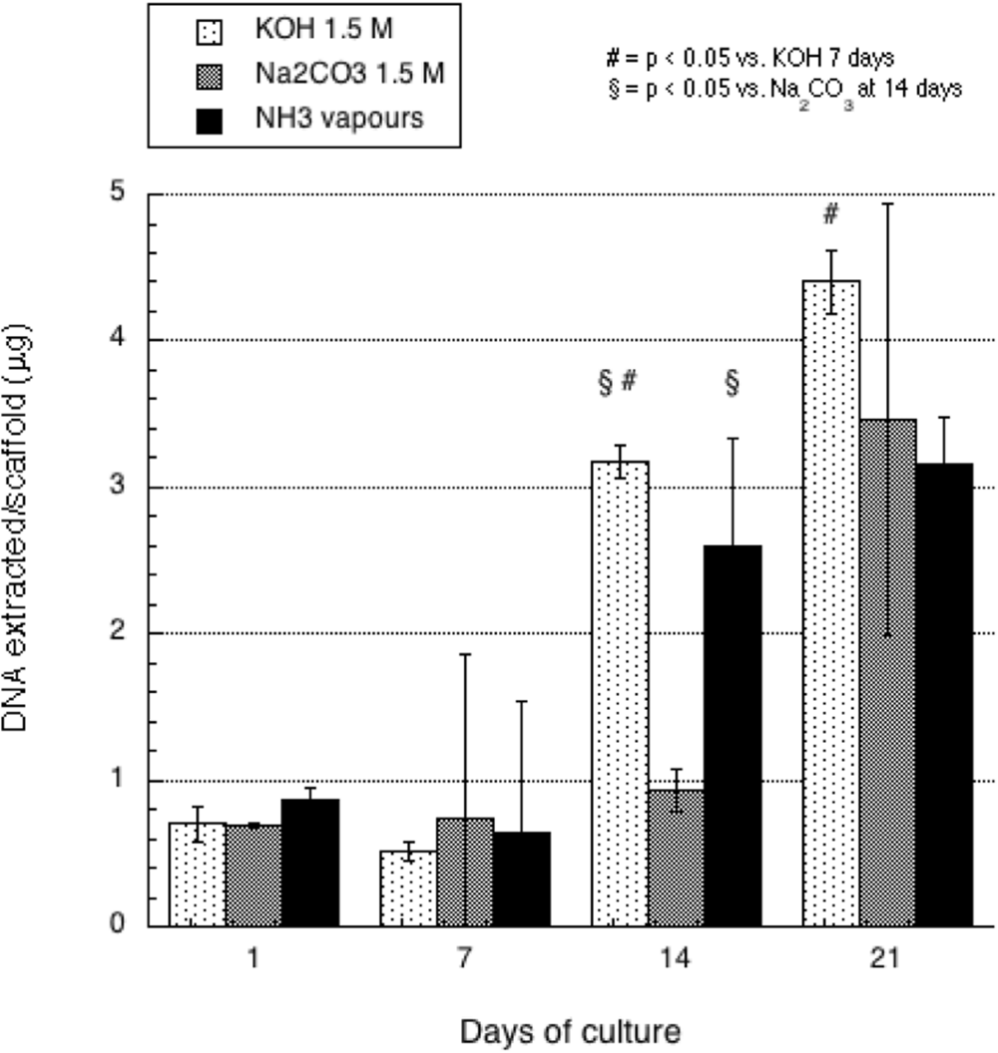
DNA assay of human fibroblasts grown on 3D printed chitosan scaffolds gelled with KOH (1.5M), Na_2_CO_3_ (1.5M), NH_3(g)_ (from 28% ammonia solution) performed at different time points. The bars represent the standard error of the mean (n= at least 3).

Cell proliferation onto scaffolds was monitored on the basis of the amount of DNA isolated at each time point over 21 days. Data in Table 2 and Fig. 6 show the growth trend of cells on scaffolds. To asses cell viability an MTT assay was performed at all time point, confirming the growth trend observed by DNA extraction (Figure S5 supplementary Material). At 14 days of culture cell proliferation on scaffolds prepared on Na_2_CO_3_ is significantly less pronounced with respect to other types of scaffolds. After 21 days of culture, cells had almost completely covered scaffold surface: scaffolds prepared with ammonia vapours show a non-significant delay in cell colonization with respect to scaffolds prepared in KOH 1.5 M. On the whole, all kinds of scaffolds adequately support cell growth, favouring cell migration and proliferation.

**Table 2:** Amount of DNA extracted per scaffold 7, 14 or 21 days after seeding on scaffolds prepared by different gelation media (mean ± standard deviation).

Gelation media	7 days	14 days	21 days
	Mean (μg) ± std.dev. (μg) per scaffold	Mean (μg) ± std.dev. (μg) per scaffold	Mean (μg) ± std.dev. (μg) per scaffold
KOH 1.5 M	0,51 ± 0,06	3,18 ± 0,12	4,40 ± 0,23
Na_2_CO_3_ 1.5 M	0,77 ± 1,08	1,07 ± 0,21	3,46 ± 1,47
Ammonia vapours	0,64 ± 0,90	3,23 ± 0,70	3,16 ± 0,33

## Discussion

The utilization of chitosan as polysaccharide for manufacturing biomedical scaffolds is highly investigated. When using 3D printing technology the precise and accurate control of designed geometries is pivotal to fabricate the desired scaffolds and correlate the 3D macro- and micro-structure to cell responses. For these reasons, CH post processing gelation should be carefully investigated to understand its 3D behaviour. Obviously, the capability to retain the frozen 3D structure is directly correlated to the time of gelation, and different parameters are involved in this two-step network-structuring process: the pH, physical state, activity and the interaction capability of the basic medium. Independently from the resulting pH, not all solutions were adequate to keep the structure conferred by the 3D printing of the scaffolds (Table 1): in particular, solutions containing sodium bicarbonate arouse bubbling (CO_2(g)_), which led to the deformation or collapse of the 3D structure (Figure S1A Supplementary Material).

KOH 1.5 M (pH 14) and 1 M (pH 14) solutions resulted in the same pH, but from our data, chitosan cross-linking resulted more efficient when the 1.5 M solution was used. This could be attributed to the higher concentration of OH^−^, which are driven by diffusion gradient to penetrate into the scaffold in less time (Table 1) leading to a more rapid CH neutralization (∼30 sec vs 50 sec): this leads to a faster formation of a stable network, better retaining the structure conferred by 3D deposition. Excellent homogeneous porous distribution both on the surface and inside the filaments was observed (Figure S3 Supplementary Material).

The use of sodium carbonate 1.5 M (pH 11.94) allowed for the rapid gelation (∼40 sec) and retention of the 3D structure. As retention of the frozen structure is a compromise between a local exothermic acid-base reaction and the neutralization reaction, in this case a partial collapse of the internal CH network was observed (Figure S3B Supplementary Material) due to the slower formation and diffusion of OH^−^ ions through the filaments. As a consequence, a reduced homogeneity of the inner porosity of the filament was observed. The highest diffusive properties of the ammonia gaseous medium (pH 13), together with the interaction properties of ammonia with chitosan have driven an extremely fast neutralization reaction (<20 sec). Excellent retention of the 3D structure and good homogeneity of the surface and inner porosity of the scaffold were observed.

On the whole, the type of gelation influences water content as well as the ability to regain water after dehydration. Experiments dehydration and rehydration were performed with the purpose of evaluating the chance to propose a fully dehydrated scaffold for clinical application. As hypothesized, used gelation techniques gave rise to distinct stable polymeric networks, slightly affecting material affinity toward water in addition to their total content. Gelation reaction in Na_2_CO_3_ and ammonia vapours is milder, probably allowing a local re-adaptation of chitosan chains as long as the gelation medium penetrates into the scaffold, resulting in a higher affinity for water, as witnessed by the results obtained by DSC about the amount of free water (Na_2_CO_3_ > ammonia vapour > KOH). This is also consistent with the differences in structure evidenced in SEM images and with higher mechanical resistance recorded on scaffold produced with KOH as a gelation medium. This can be further supported by the fact that the overall contraction of the structure after dehydration was greater for scaffolds prepared with KOH, thus probably offering a smaller surface area for water to get into the scaffold in the rehydration phase.

Since the complete dehydration of scaffolds leads to an alteration of their characteristics, a partial dehydration (20–40% dehydratation) is considered anyway preferable to the fully hydrated scaffold in view of a clinical application: actually a partially dehydrated scaffold could act more efficiently when applied on a wound in absorbing exudates or fluids, positively contributing to stop bleeding.

In regenerative medicine the quantification of free and bound water can be helpful to check the material similarity with respect to an autologue tissue. As mentioned, free water acts as solvent for proteins and several other compounds and, due to the fact that developed chitosan based scaffolds should mimic the native ECM, being able to control this parameter could help in maintaining the physiological turnover of compounds secreted by cells such as growth factors and cytokines. Chitosan scaffolds hydrogelled by immersion in the 1.5 M KOH solution contained a higher amount of bound water that was significantly different from scaffolds prepared with other two gelation techniques considered (11.43 ± 4.23% in KOH of bound water vs ∼4.4% average in carbonate or ammonia). Human body tissues contain more than 10% of bound water^20^, and the differences in free and bound water here evidenced could be exploited for different applications of hydrogels, choosing the most appropriate one on the basis of the target area of the body and/or for the addition of active compounds,

In tissue repair the values of elastic moduli play a pivotal role. The results observed in this work demonstrated that the three selected gelation media allowed to obtain 3D structures with comparable elastic moduli having values comparable to those observed in skin, in particular in volar forearm region (Liang and Boppart^21^. This suggests that if applied as wound healing patch, this could be integrated into wound and adapt to it.

We have previously demonstrated^14^ that chitosan-based 3D-printed scaffolds were characterized by a good interaction with cells and by an increased capacity to allow cell growth, compared to scaffolds obtained by pouring the solution on a cast^8^. In this work, we further find that all the three mild conditions tested for scaffold gelation ensure an efficient cell attachment and proliferation. The results obtained with the scaffolds gelled with Na_2_CO_3_ appear consistent with a previous work^22^, while the approaches based on KOH and NH_3_ were explored here for the first time. As clearly suggested by the fluorescence images, the complete covering of the scaffold surface observed after 14 days of cultures derives from both growth of cells directly plated onto the scaffolds and from migration from the well. This behaviour is consistent with previous data from our group on the ability of chitosan hydrogels, to support cell growth^8^ and is probably a result of the entrapment and possible retain into the structure of chemotactic and growth factors deriving from serum. All the saffolds were able to retain their structure for months when stored in water, saline solution, or in airtight environment after partial dehydration: in this case no statistically significant alteration of the structure occurred, up to six months (p > 0.05). When immersed in culture media all scaffolds retained their original dimensions up to 21 days, but the structure becomes softer and loses its consistency in about 7 days, preventing an estimation of residual mechanical resistance.

## Conclusions

3D chitosan scaffolds with appropriate size, morphology, water content and mechanical properties can be prepared *via* a simple and reproducible 3D cryoprinting technology. Three different gelation media having pH values greater than 12, i.e. KOH 1.5 M, Na_2_CO_3_ 1.5 M and ammonia vapours resulted to be suitable to accurately retain the 3D printed structure starting from the CAD design (nominal filament diameter was 250 μm). At the maximum swollen state the structure accuracy was 101% for the scaffold gelled with the ammonia vapors, 109% for those in KOH and 117% for those in Na_2_CO_3_ (RSD < 10%). The physical properties and stability of these systems in biological media mostly depended on the gelation media and are a balance between chitosan network interactions and scaffold water content. The scaffold gelled in Na_2_CO_3_ resulted in highest free water content (96.78 ± 2.47%). The dehydration/rehydration experiments exhibited that scaffolds gelled in Na_2_CO_3_ and ammonia can be dehydrated up to 60% for storage, as they are able to reach the complete hydration condition after immersion in aqueous solution. The scaffolds prepared in KOH can be dehydrated up to 80% without losing they properties. No complete dehydration is allowed without irreversibly compromise the 3D structure. As for mechanical resistance scaffolds prepared with ammonia vapours showed a significantly higher elastic modulus with respect to both other media. All scaffolds were able to enhance and drive cell growth up to 21 days and this result has important therapeutic implications, since an efficient migration of cells into scaffolds is essential to guarantee the quality of tissues regeneration.

## Methods

### Materials

Chitosan ChitoClear™ TM4030, having a degree of deacetylation of 75% and a molecular weight of 50 kDa was obtained from Primex Ehf (Iceland) and used as received. Acetic acid and D-(+)-raffinose pentahydrate were obtained by Sigma-Aldrich, USA. All salts were from A.C.E.F. (Italy), while ammonia solution 28% was from VWR International (USA). All solutions were prepared using ultrapure water obtained with a Purelab Flex 1 system by ELGA Veolia.

### Preparation of chitosan solution and 3D printing

Chitosan Chitoclear was dissolved in ultrapure water at 6% w/v concentration with the help of 2% v/v of acetic acid under stirring. As previously described by Bettini *et al*.^8^, after complete dissolution of chitosan, D-(+)- raffinose pentahydrate was added at 290 mM final concentration as a viscosity agent and stirred until complete dissolution. (For the determination of neutralization process in different gelation media described below bromothymol blue was added as a pH indicator). The resulting solution was loaded into a 10 ml syringe that was accommodated on a 3D printer built in-house^14^. Briefly, the machine is based on a three cartesian axes system that allows the movement on the horizontal plane of the printing plate and the vertical translation of the nozzle. Chitosan solution in the syringe is extruded by a pump acting on a syringe mounting a 26 G needle, deposed, layer by layer, on the printing plate at a velocity of 3 mm/s and instantaneously frozen to keep the structure of the scaffold conferred by the software. Scaffolds were shaped as a net of 1.6 cm × 1.6 cm in size, composed by the alternation of 10 or 20 orthogonal layers composed of parallel filaments being 200 μm distant from each other.

### Gelation media

After freezing of the 3D printed scaffold, the structure was fixed by contact with a basic environment, in liquid or gaseous state. In order to obtain mild gelation conditions, different solutions were prepared in ultrapure water to obtain gelation of the scaffolds by immersion: the composition of the solution and the corresponding tested is reported in Table 1. As an alternative method for gelation, frozen scaffolds were transferred to a chamber saturated with ammonia vapor and exposed to the gas.

The effect of time of exposition to different gelation media was evaluated on the behavior of scaffolds in terms of water content, ability to recover water after dehydration: in particular gelation was evaluated after exposition to different media for 3, 15, 30, 60 minutes, 3 hours or 24 hours.

### Evaluation of water content and water recovery after dehydration

Ten-layers scaffolds produced with selected methods of gelation were further analysed for water content and ability to absorb water after total or partial drying. After gelation, every scaffold was washed twice by immersion in ultrapure water for ten minutes; then surface water was removed by positioning each scaffold on a Buchner funnel covered by filter paper and by applying vacuum for 20 seconds. Time points checked were 3 min, 15 min, 30 min, 1 h, 3 h and 24 h. The wet weight of each scaffold was measured and registered as W_0_. The dehydration was performed by leaving each scaffold in vacuum oven (Gallenkam) at 40 °C and 200 mbar for 30 and 60 minutes and by recording the weight at each time point. All weights were compared to the dry weight (W_d_) of scaffolds, which was obtained by lyophilisation for 24 h in a Christ Alpha 2–4 LSC plus Freeze-Dryer. For each gelation medium and time point the experiment was repeated three times.

This first experiment gave us information about the time needed to obtain a specific degree of dehydration for each gelation medium. This was selected to obtain a residual 30%, 45%, 60% or 80% in weight of total water content. These partially dried scaffolds were immersed in ultrapure water to evaluate the ability to recover initial water content in 30 minutes, 1, 3 6 or 24 hours.

### Differential scanning calorimetry analysis

Differential scanning calorimetry (DSC) has been chosen to evaluate the amount of bound water in chitosan-based scaffolds as a function of gelation with the conditions tested.

Once produced, scaffolds underwent the reaction in the three different gelation media (KOH 1.5 M, Na_2_CO_3_ 1.5 M, ammonia vapors) for 1 hour and then were washed twice for 10 min in ultrapure water (UP) as in the standard procedure previously described. A Buchner filtration has been assessed for 20″ on filter paper to remove the excess of water present within macropores and a 4 mm diameter stamp has been used to obtain identical specimens.

DSC measurements were performed using an Indium calibrated (onset of melting Tm = 156.48 C, enthalpy of melting DHm = 28.60 J g1) DSC 821e instrument (Mettler Toledo, Switzerland) driven by STARe software (Mettler Toledo). DSC traces were recorded by placing accurately weighed quantities (6–12 mg) of frozen samples in a 40 µL aluminium pan which was sealed before the weightings to avoid water evaporation. Scans were performed between −7 and 35 °C at a scanning rate of 10 °C min^−1^ under a purging nitrogen atmosphere (100 mL min^−1^). Each measurement was carried out at least in triplicate. Data relevant to the observed thermal events were reported as peak temperatures.

Total water amounts in the scaffolds have been determined by gravimetric analysis: 10 of each scaffold types (gelled by different alkaline media) at 100% of hydration have been weighted after 20″ of Buchner filtration and re-weighted after O.N. drying in a Thermocenter TC100 (Salvislab) oven at 40 °C. Total water content was thus calculated by means of the formula: $(Wh\, \mbox{-} \,\overline{Wd})$; where “*h*” stands for “hydrated” and “*d*” for “dehydrated”.

Since only bulk water freezes and melts completely, it has been quantified integrating peaks of the endothermic thermal events of each scaffold, obtaining the amount of energy (Joules) required to melt free water in the sample.

In order to accurately calculate free water quantities in the samples a calibration has been priorly assessed analysing with the same procedure known UP water quantities. This allowed to establish a mathematical correlation between a determined free water quantity and the required energy to melt it; a calibration curve with the function [y = −437.18x] has been drawn.

Melted water in the samples was then quantified and finally reported as percentage of free water compared to the total content initially calculated.

### Mechanical resistance

The mechanical resistance of scaffolds obtained by different gelation media was compared on 20-layers scaffolds having size of 5 cm × 1.5 cm. Hydrogels at the maximum swollen state were tested. Thickness was determined as a mean of six measurements of the scaffold performed with a digital micrometer (Mitutoyo, Japan). Each scaffold was fixed on a tensile tester (AG M1 Acquati, Italy) loaded with a 5 daN cell. Force and time signals were digitalized by means of a PowerLab 400 board and recorded with Scope 3.5 software. Elongation at break (% strain) and Young’s modulus were determined from the relevant stress-strain curves, taking into consideration the linear portion.

### SEM analysis

Scaffolds obtained with different gelation media underwent critical point drying, then images were taken with a scanning electron microscope (Sigma HD, Carl Zeiss, Jena, Germany), at 300X magnification and EHT 1.00 kV. All images were analysed by ImageJ software (NIH, Bethesda USA) for the measurement of macro- and micro-structures, mean pore size and distribution.

### ATR FT-IR spectroscopy

ATR FT-IR was used to observe any structural modification of scaffolds due to gelation. Spectra were taken by a Nicolet 5700 (Thermo Scientific, USA) in the range 400–4000 wavenumbers (cm^−1^) with a resolution of 30 scan per second. Raw chitosan powder was analysed as a reference respect to 10 layers scaffolds produced by contact with KOH 1.5 M, Na_2_CO_3_ 1.5 M or ammonia gas and dehydrated by lyophilisation.

### Cell culture

Primary human skin fibroblasts coded as C84 were isolated with informed consent from an underarm explant from a healthy donor (female, 45-years old) and cultured, as previously described by Elviri *et al*.^14^ before seeding them on scaffolds which underwent gelation in KOH, Na_2_CO_3_ or ammonia vapours. After gelation, scaffolds were cut at a diameter of 6 mm, sterilized with ethanol 70% for 18 h, washed three times with sterile PBS, conditioned with complete medium for 30 minutes and then seeded in 48 well-plates. No scaffolds drying was performed before plating. Cells at passage 12 were harvested from the flasks at the confluent state by incubating it in trypsin solution for 2 min at 37 °C, then resuspended with DMEM supplemented with penicillin-streptomycin (100 U/mL), non essential amino acids, and serum (10%), counted and plated at density of 2 × 105 cells/well: 10 µl of cell suspension was initially seeded on scaffolds to facilitate adhesion for about 1 hour; successively, growth medium was added in order to completely cover their surfaces. The medium was changed every three days and evaluations of cell viability and microscopic analyses were performed after 7, 14 and 21 days.

### Microscopic analysis

Empty and cell-seeded scaffolds, after washing 1x with PBS to remove dead cells, were incubated with calcein-AM, a probe that becomes fluorescent and is retained inside cells in response to the activity of intracellular esterases. Such treatment was performed in growth medium at 37 °C and 5% CO_2_ for 30 minutes. Excess calcein was then removed by washing twice with PBS and images were captured using a Nikon eclipse TE300 inverted fluorescence microscope. At least two images were analysed per scaffold at each time point, using empty scaffolds as controls. Three scaffolds per group were analysed at each time point.

### Cell viability and proliferation

Cell viability and proliferation onto scaffolds was evaluated by measuring the DNA content by adaptation of a method from Hoemann *et al*.^23^. Briefly, at each time point six scaffolds for each type were harvested, pooled into a 1,5 ml eppendorf washed with PBS, and 300 µl of a 3 mg/ml papain (Sigma-Aldrich, USA) solution were added. Digestion was performed overnight at 65 °C then the resulting suspension was centrifuged and 50 µl of supernatant were transferred in a 96-well dark plate (Costar) and mixed with 200 µl of Hoechst 33258 solution. Samples were measured with a Spark® Tecan fluorimeter with excitation wavelength of 360 nm and absorption wavelength of 460 nm. DNA content onto each scaffold was estimated by interpolation from a standard curve obtained with sperm salmon DNA (Sigma Aldrich, USA) at known concentration. Viability was also assessed with the MTT assay as previously described^14^. In both assays, the absorbance obtained from correspondent empty scaffolds was subtracted from each measurement. Three scaffolds per group were analysed at each time point.

### Statistical analysis

Statistical analysis was performed with Microsoft Excel and Graph Pad-Prism software version 5.0. Data obtained with the three scaffolds types were compared with One-way analysis of variance and the Dunnett’s Multiple Comparison Test. Significance was defined as p < 0.05.

## Electronic supplementary material

Supplementary material
