# Supplementary material for: Study of 3D-printed chitosan scaffold features after different post-printing gelation processes
Source: Sci Rep. 2019 Jan 23;9:362. doi: 10.1038/s41598-018-36613-8 (PMC6344587; doi:10.1038/s41598-018-36613-8)

Study of 3D-printed chitosan scaffold features after different post-printing gelation processes

Carlo Bergonzi[1], Antonina Di Natale[1], Francesca Zimetti[1], Cinzia Marchi[1], Annalisa Bianchera[2], Franco Bernini[1], Marco Silvestri[3,4], Ruggero Bettini[1], Lisa Elviri[1]

[1]Food and Drug Department, University of Parma, Parco Area delle Scienze 27/A, 43124, Parma, Italy.

[2]Interdepartmental Centre Biopharmanet-Tec, University of Parma, Parco Area delle Scienze 27/A, 43124, Parma, Italy.

[3]Department of Engineering and Architecture, University of Parma, Parco Area delle Scienze 181/A, 43124, Parma, Italy

[4]Department of Innovative Technologies, University of Applied Sciences and Arts of Southern Switzerland (SUPSI), CH-6928, Manno, Switzerland.

AUTHOR INFORMATION

Corresponding Author: Lisa Elviri

Phone: +39 0521 905087

Fax: +39 0521 905006

E-mail: lisa.elviri@unipr.it

Figure S1. Neutralization process (after 1 min) and 3D retention structure observation by the colour changes in printed scaffolds from a chitosan solution to which blue bromothymol was added as a pH indicator.

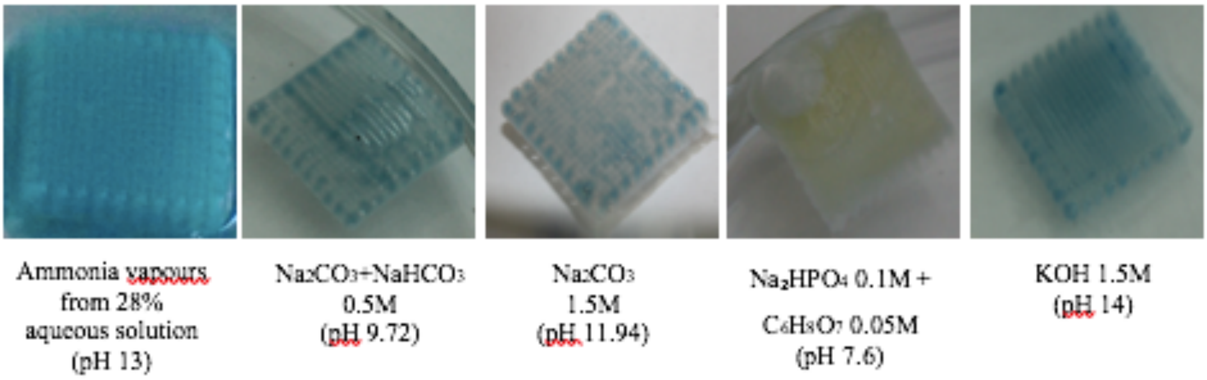

Figure S2. Stress-strain curve of the 3D printed chitosan scaffolds gelled in KOH 1.5M, Na_2CO_3 1.5M and ammonia vapours.

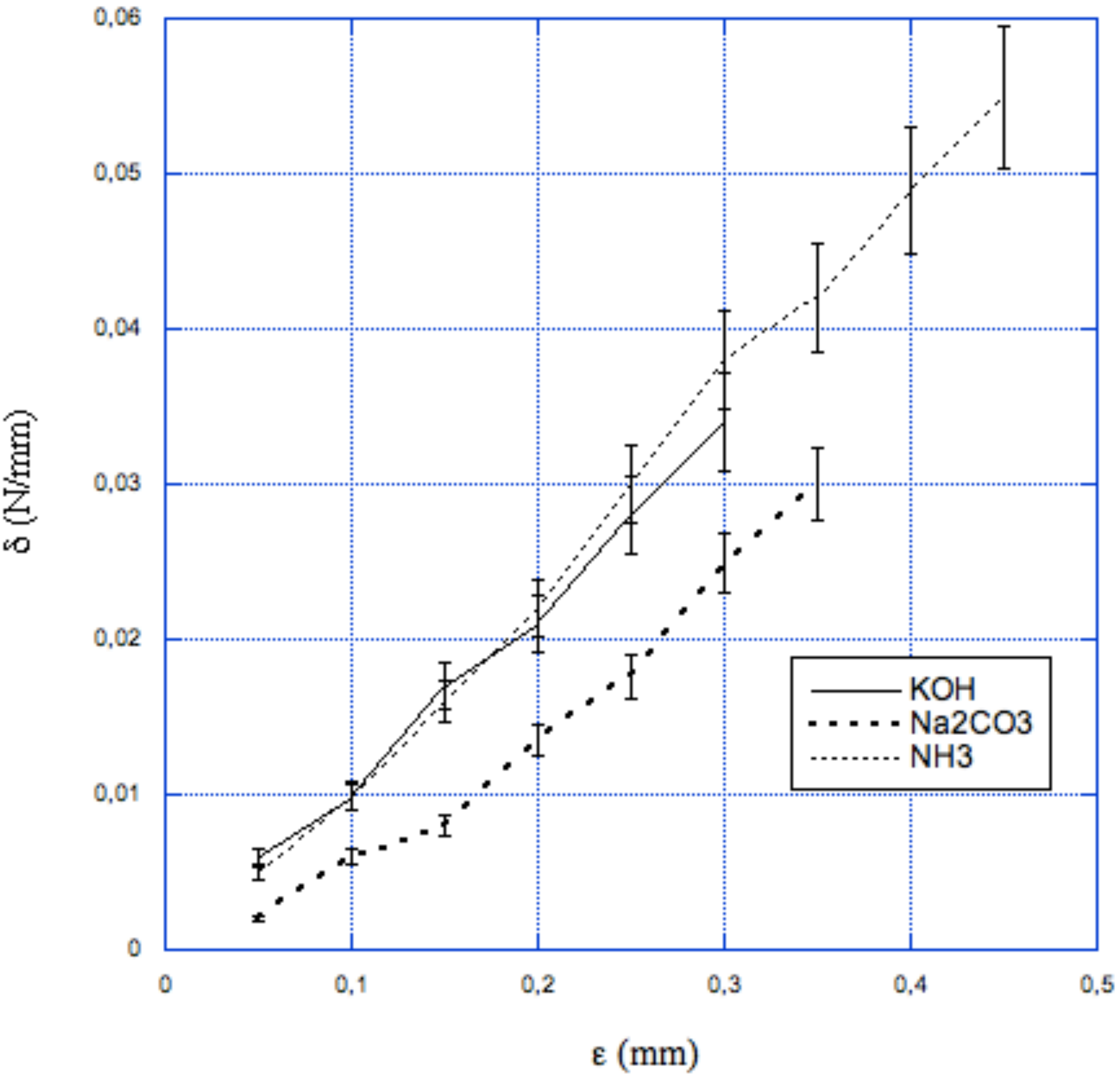

Figure S3. SEM images of printed chitosan scaffolds (filament surface and cross-section) processed in three different gelation media (A) KOH 1.5M; (B) Na_2CO_3 1.5M; (C) ammonia vapours. (500X magnification).

(A)

(B)

(C)

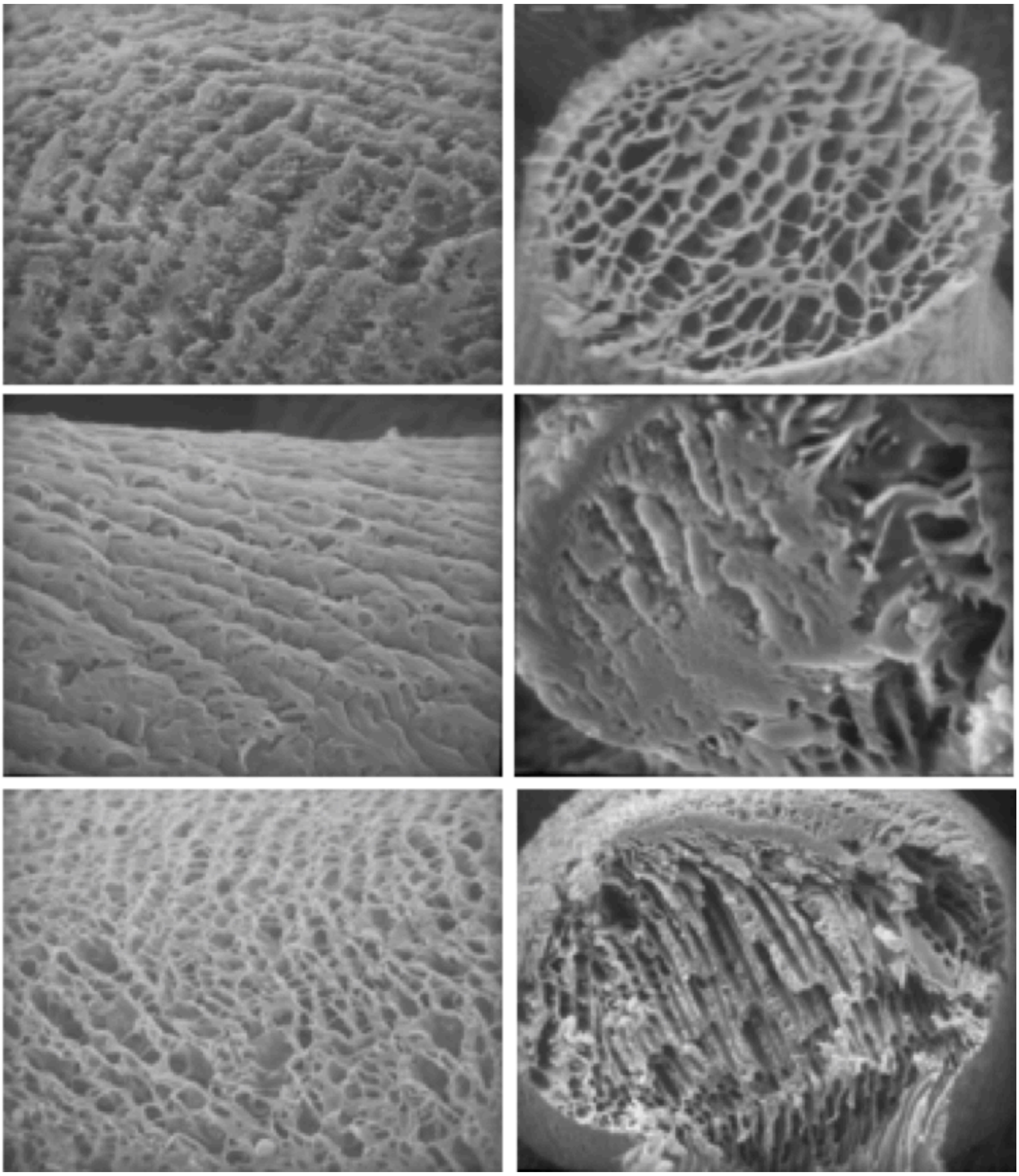

Figure S4. ATR FT-IR spectra of raw chitosan compared with spectra of hydrogels prepared with the selected gelation medium (gelation time 1 min).

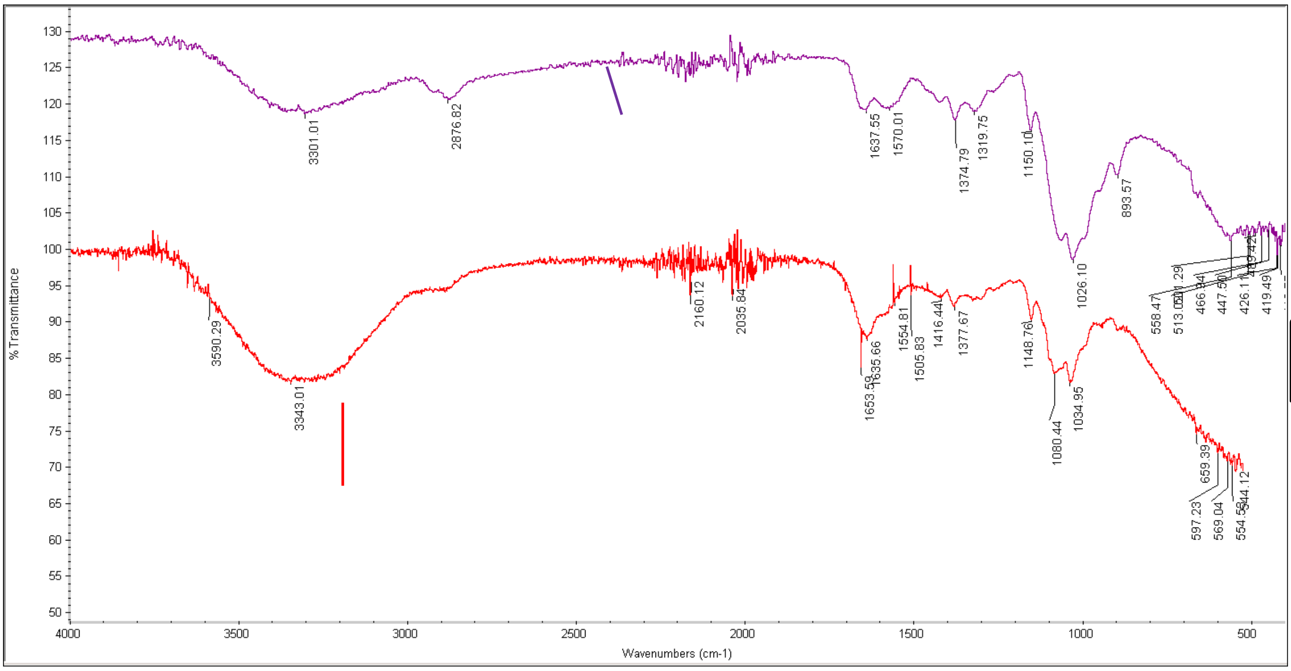

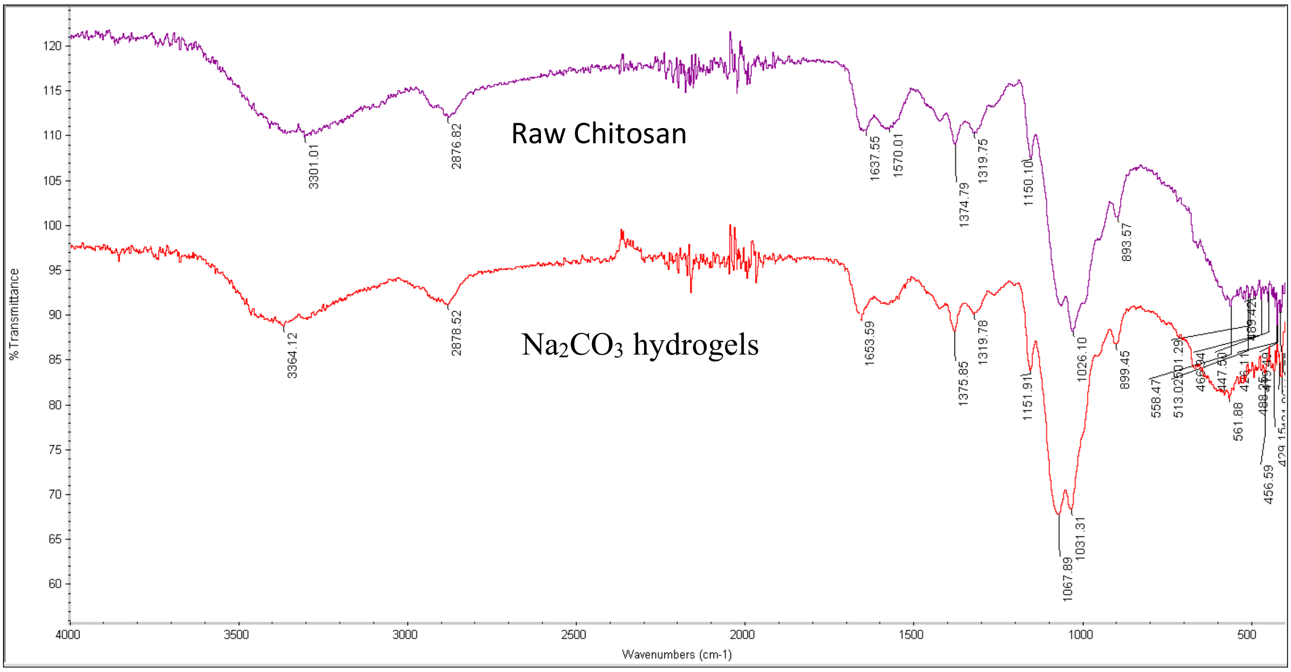

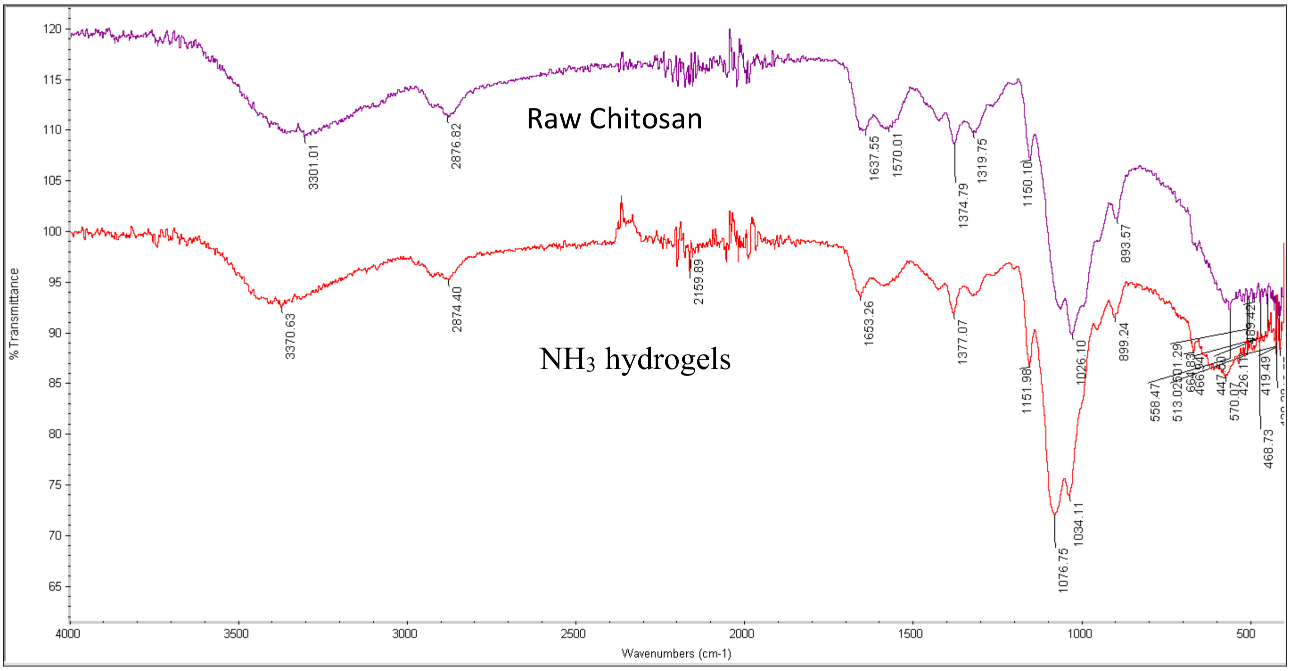
Raw Chitosan
NH3 hydrogels
% Transmittance
Wavenumbers (cm-1)
3301.01
2876.82
1637.55
1570.01
1374.79
1319.75
1150.10
893.57
3370.63
2874.40
2159.89
1653.26
1377.07
1151.98
1026.10
899.24
1076.75
1034.11
558.47
468.73
419.49

Figure S5. MTT assay of human fibroblasts grown on 3D printed scaffolds gelled with KOH (1.5M), Na_2CO_3 (1.5M), $NH_{3(g)}$ (from 28% ammonia solution) performed at different time points. The bars represent the standard error of the mean (n= at least 3).

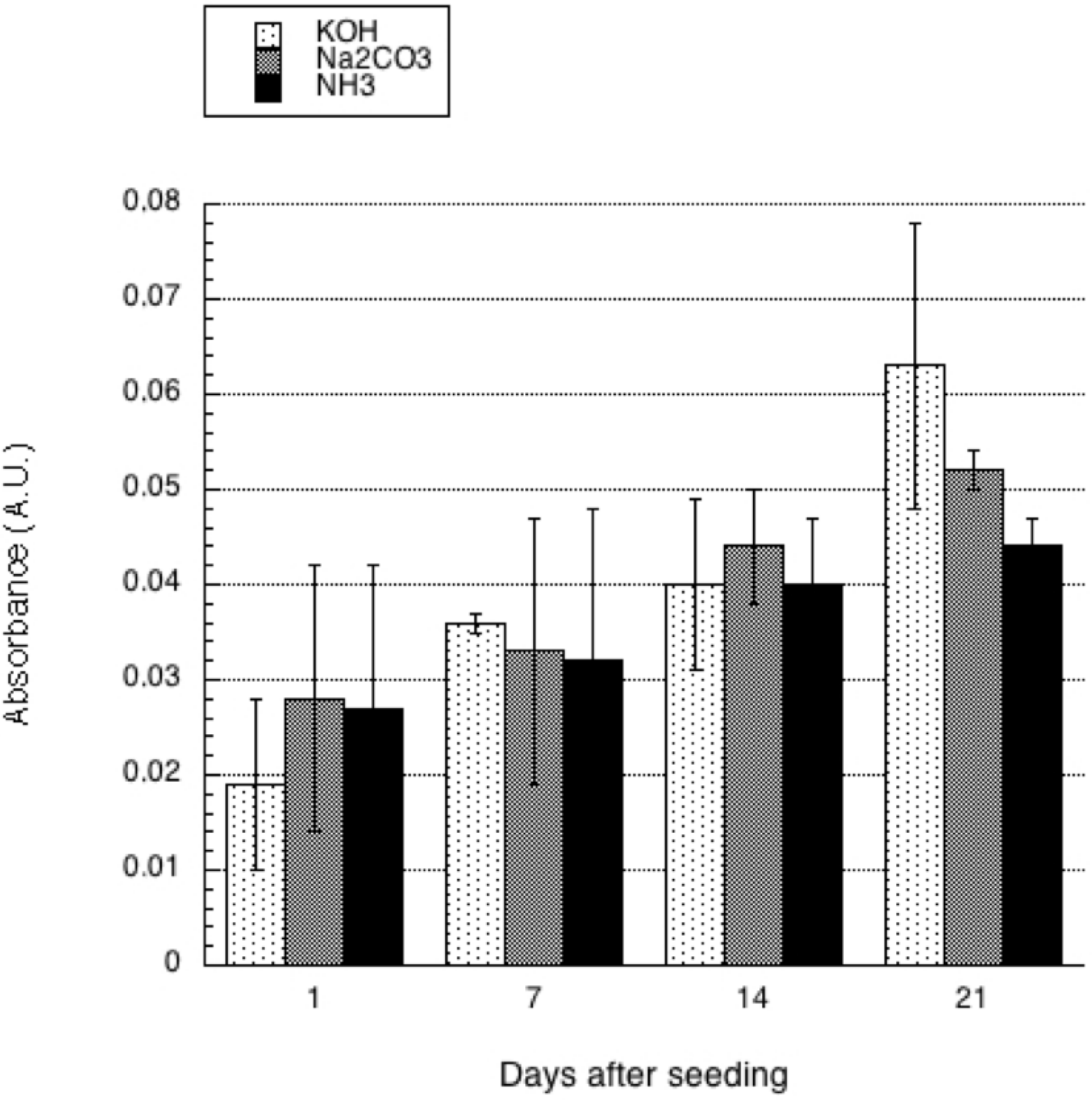

Supplement: Supplementary file 1 — Supplementary material [file 41598_2018_36613_MOESM1_ESM.pdf]
